# Effective Prediction and Important Counseling Experience for Perceived Helpfulness of Social Question and Answering-Based Online Counseling: An Explainable Machine Learning Model

**DOI:** 10.3389/fpubh.2022.817570

**Published:** 2022-12-22

**Authors:** Yinghui Huang, Hui Liu, Shen Li, Weijun Wang, Zongkui Zhou

**Affiliations:** ^1^School of Management, Wuhan University of Technology, Wuhan, China; ^2^Key Laboratory of Adolescent Cyberpsychology and Behavior, Ministry of Education, Wuhan, China; ^3^School of Psychology, Central China Normal University, Wuhan, China; ^4^Central China Normal University Branch, Collaborative Innovation Center of Assessment for Basic Education Quality at Beijing Normal University, Wuhan, China; ^5^School of Music, Henan University, Kaifeng, China; ^6^Institute of Digital Commerce, Wuhan Technology and Business University, Wuhan, China

**Keywords:** perceived helpfulness, social question answering, online counseling, explainable machine learning, topic consistency, linguistic style similarity, emotional similarity

## Abstract

The social question answering based online counseling (SQA-OC) is easy access for people seeking professional mental health information and service, has become the crucial pre-consultation and application stage toward online counseling. However, there is a lack of efforts to evaluate and explain the counselors' service quality in such an asynchronous online questioning and answering (QA) format efficiently. This study applied the notion of perceived helpfulness as a public's perception of counselors' service quality in SQA-OC, used computational linguistic and explainable machine learning (XML) methods suited for large-scale QA discourse analysis to build an predictive model, explored how various sources and types of linguistic cues [i.e., Linguistic Inquiry and Word Count (LIWC), topic consistency, linguistic style similarity, emotional similarity] contributed to the perceived helpfulness. Results show that linguistic cues from counselees, counselors, and synchrony between them are important predictors, the linguistic cues and XML can effectively predict and explain the perceived usefulness of SQA-OC, and support operational decision-making for counselors. Five helpful counseling experiences including linguistic styles of “talkative”, “empathy”, “thoughtful”, “concise with distance”, and “friendliness and confident” were identified in the SQA-OC. The paper proposed a method to evaluate the perceived helpfulness of SQA-OC service automatically, effectively, and explainable, shedding light on the understanding of the SQA-OC service outcome and the design of a better mechanism for SQA-OC systems.

## Introduction

Throughout the world, people are affected by mental health disorders at staggering rates (1). In many contexts, appropriate treatment is lacking and people with mental health conditions experience severe human rights violations, discrimination, and stigma (2). Moreover, there are direct and indirect consequences of COVID-19 on mental health conditions, which challenged traditional mental health systems, leading to increased demand and interrupted delivery of essential services at the same time.

With the rapid development of the mobile internet and the extensive practice of the concept of “Internet Plus”, online counseling service has become an emerging market (3) and a large number of online counseling apps have emerged (4). Scholars have defined online counseling as the delivery of counseling services via the Internet when the pastoral/spiritual counselor or psychologist and counselee are not in the same physical area and communicate using computer-mediated communication innovations (3, 5, 6). Online counseling encompasses a wide range of techniques, including but not limited to instant messaging, synchronous chat, text messaging, video conferencing, and asynchronous email (7). There has been evidence showing that the break out of COVID-19 has boosted the use of online counseling worldwide (7–9). Still, access to mental health care remains a global challenge with widespread shortages of the workforce (10). Facing limited in-person treatment options and other barriers like stigma (11), millions of people are turning to SQA-OC platforms such as TalkLife (talklife.co), OnePsychology (xinli001.com) to express emotions, share stigmatized experiences, and receive peer support or volunteerism from the counselors (12).

One of the major information and knowledge sources that have arisen from Internet-mediated social practice is social questioning and answering (SQA) sites. People use social question answering sites for expressing their knowledge demands (13–15), seeking information (16, 17) as well as for the construction and maintenance of relationships (18). SQA, as an example of collective intelligence, allows users to pose questions, contribute answers and comments, and evaluate questions and responses among peers. Moreover, compared with the privacy and anonymity of face-to-face counseling, SQA-OC avoids privacy and ethical issues in the counseling process, which can greatly enhance the initiative, accessibility, and immediacy of psychological counseling services and is in line with epidemic prevention policies such as social isolation caused by COVID-19. However, while peer supporters on these platforms are motivated with the intention of helping others seeking support (henceforth seeker), most of them are not well-trained and typically lack the knowledge of best practices in the therapeutic process. Such informal help has been demonstrated to be more comfortable for the public but less impactful than formal counseling services (19). As the growing relevance of social Q&A platforms for psychological counselees, there is a large and urgent need for evaluating and forecasting the content quality of the counselor's responses. Specifically, what influences the counselees' perception of counselors' service quality in SQA-OC and how various factors interact remains unclear.

This study will introduce the notion of perceived helpfulness to measure the extent to which SQA-OC platforms help the public. The notion of perceived helpfulness has been widely used in the study of online consumers (20, 21), which refers to the degree to which consumers feel useful to online information or services and is usually measured by the total number of helpful notes voted by consumers (22–24). This voting mechanism brings huge revenue to the commercial platform (25), as well as filter out valuable information for consumers when faced with massive options. This success of voting helpful online services has been applied to the SQA-OC platform, by providing counselees with voting options for whether or counselors' responses are useful, and the public including counselees can check the number of peer votes to analyze the counselor helpfulness. This voting mechanism makes useful responses from the counselor stand out from an SQA-OC platform and helps counselees and the public make decisions (22, 26). Therefore, we applied the notion of perceived helpfulness to measure the outcome of SQA-OC in this study, as the total number of helpful notes becomes an intuitive standard for measuring and evaluating the service quality of counselors on the online platform.

In formal and face-to-face counseling, the quality of counseling service is mostly measured via interviews and questionnaires, during which the counseling competence, counseling strategy, and verbal skills of the counselor are assessed (27). More specifically, the investigation into the talking and conversations in the counseling process becomes an important way to measure counseling outcomes (28). Benefiting from the development of natural language process (NLP) and the tool of language inquiry and word count (LIWC), the linguistic features of counselors and counselees can be studied in a large scale and quantitative manner. Using NLP analysis, the existing research found that the change of counselees' linguistic features (e.g., first-person use, negative emotion words) can positively predict the therapeutic effect (29–31). In addition, there are significant differences in the linguistic features (e.g., positive/negative emotion words, causation words) between good counselors and poor counselors (32, 33).

In terms of the counselor-counselee interaction, several scholars used a computational approach to measure empathy in the text (34) and the counselor-counselee emotional similarity (35). According to Ardito and Rabellino (36), the emotional connection between the counselor and the counselee is closely related to the working alliance and the evaluation of therapy outcomes. In the field of commercial research, language style matching (LSM) (37) between the manager and the reviewer has been demonstrated to be an important factor in predicting the public's perceived helpfulness (38). Using the same approach, LSM between the counselor and the counselee has been proved to be effective in predicting the counseling outcome (35). However, the above studies that employed NLP methods mainly focused on the formal and synchronized counseling process, yet little is known about the informal and asynchronous online SQA-OC context. Therefore, this study analyzed the linguistic cues and psychological topics from SQA-OC, build a model to predict and explain the perceived helpfulness of counselors' response effectively, as well as identify helpful counseling experiences. This research aims to address the following two questions:

Research question 1: Dose linguistic cues and machine learning methods can be utilized to predict the perceived helpfulness of SQA-OC effectively?
Research question 2: How do linguistic cues contribute to the perceived helpfulness of SQA-OC?

In recent years, the SQA-OC has shown explosive growth, but its quality is uneven (39) which makes it difficult to monitor by manpower. By using the machine learning method, this study aims to propose an automatic detection approach to measure and understand the counseling outcome and service quality of SQA-OC. The method can reduce large amounts of questionnaire evaluation or qualitative analysis processes while providing feedback for the platform and the online users more quickly and efficiently on a large scale. Likewise, by proposing NLP and XML methods, this study aims to identify influential linguistic cues and their impact on perceived helpfulness, to advance ours understanding of SQA-OC outcome, which could afford manipulable and real-time feedback to the counselors and SQA-OC platform.

## Data, Methods, and Measures

### Data Crawling

Data were crawled from one of the largest Chinese online counseling platforms “One Psychology Community”,^1^ on which nearly 20 million asked for psychological help. In the Q&A section of the platform, psychological seek-helpers could post their psychological distress and problems, seek psychological service and support from the platform's counselors anonymously. We used “bazhuayu”,^2^ a web scraping software, to crawl 5,169 questions from the counselee, as well as 15,058 responses from the counselor. The time span of the SQA-OC data is from June 17, 2013, to December 16, 2020. A report conducted by a famous Chinese online counseling platform, “JianDanXinLi”^3^ showed that among the users of online counseling, the female visitors were more, who were three times more than the male visitors, and visitors in the early adulthood (21–35 years old) accounted for 77.57%.

Each question has the following three sections: the title of the description, the description of the psychological problem, and the asking time, which contains an average of 185 words. Questions may include components such as the title of the post, age, gender, course of the psychological problem, inner feelings, duration of the problem, and the label (i.e., occupation, marriage, romantic relationship, family, etc.). These questions are followed by several responses from counselors, which contains an average of 388 words. The number of users' likes given to the counselor ranges from 1 to 39, with an average of 4.362.

### Word Embedding Based Psychological Topic Detection

Word embedding is a popular machine learning method that represents each word by a vector, such that the geometry between these vectors captures semantic relations between the corresponding words. Since it was demonstrated that word embedding can encode rich semantic relationships between words as geometrical relationships in low-dimensional vector space (40, 41), the embedding models have offered novel opportunities and solutions to challenging problems, including language evolution (42), gender and stereotypes (43, 44), culture and identities (45, 46), and even the prediction of material properties (47). For the analysis of psychological topics of counselees and counselors, word embedding was utilized to extract the symptoms and influencing topics from the SQA texts.

According to former researchers which applied word embedding to identifying psychological topics in online psychological help seeking texts (9, 48), we proposed following four steps. Firstly, a predefined lexicon regarding psychological symptoms and influential linguistic cues of was constructed. The seed words of the lexicon were extracted by two Ph.D. candidates in psychology from three text resources: Kessler 10 and Patient Health Questionnaire (49) and the question tag system^4^ of One Psychology website. Secondly, we built the psychological lexicon of the SQA-OC community. By using the Jieba tool (i.e., a Python segmentation package for Chinese^5^, and Baidu stop-word list, the text of SQA-OC was cut, and stop words were deleted. According to the word embedding method, the text was used as the training corpus. The word embedding method of Word2vec in Gensim software^6^ was used to construct the latent semantic model for large-scale SQA text, to obtain domain lexicons of psychological symptoms and influencing factors, respectively. Specifically, the cosine similarity between the words in the model vocabulary and the predefined lexicon was calculated based on the model. Two graduate students were recruited to set the thresholds of cosine similarity to remove words in SQA text which were irrelevant to the predefined vocabulary, to checked the retained words manually. Specifically, the psychological lexicons contain two parts: 2,567 words related to psychological symptoms and 1,077 words related to psychological factors.

The third step was to obtain topics of the psychological symptoms and influential linguistic cues of the counselees and counselors. According to the lexicon we built, the psychological words from the SQA-OC text were selected. Using the average word embedding method, word vectors representation of symptoms and influential linguistic cues for counselees' questions and counselors' responses was obtained (50). We used the k-means algorithm (python implement in scikit-learn) and its evaluation index (i.e., silhouette coefficient), to obtain and evaluate the clustering performance with different numbers of clustering centers. Fourth, we selected the best k-mean clustering model for topics detection. Then, the number of cluster under the optimal silhouette coefficient was selected to construct the clusters of psychological symptoms and influential factors. Finally, the topics related to psychological problems are named as depression and anxiety, suffering, social phobia, lack of interest, suicidal tendencies, worry (afraid), and anger. The topics related to influential linguistic cues are named as love, marriage, psychotherapy, work, interpersonal relationship, character, and family (see Table A2 in Appendix for detailed topic information).

### Measures

#### Dependent Variable: Perceived Helpfulness of Counselor's Responses

One psychology platform provides the counselees with voting opportunities for whether a counselor's responses are useful or not. Owning to the anonymity of the platform, the questions and answers are visual to the public, a counselor's responses can be voted as useful or not (i.e., a binary measure of helpfulness) by both the counselee and others who are browsing the questions. We selected the number of helpful votes to measure perceived helpfulness, which is in line with previous studies of online (22–24, 27–35, 37, 38).

#### Explanatory Variable 1: Linguistic Cues in Text From Counselors and Counselees

##### Linguistic Cues in Counselees' Text

Given that the question of counselees in the One Psychology platform is visible to the public^7^; other counselees may vote the counselor's responses as useful or not after reading one counselee's question. Therefore, we hypothesized that the linguistic cues in the counselees' text will influence the perceived helpfulness of SQA-OC.

##### Linguistic Cues in Counselors' Text

Previous studies have found that the therapeutic outcome can be predicted from the linguistic cues on counselors' use of language (32, 33). We hypothesis that the linguistic cues in counselors' responses will influence the perceived helpfulness of SQA-OC.

For the linguistic cues measures of either the counselors' text or the counselees text, we used the Simplified Chinese version of LIWC (SCLIWC) to extract the linguistic cues including affective processes (AP), social processes (*SP*), cognitive processes (*CP*), perceptual processes (*PP*), biological processes (*BP*), Drives (*Dr*), time orientations (*TO*), relativity (*Rev*), personal concerns (*PC*), and informal language (*IL*). We also included the number of words, word per sentence, number of sentences, number of function words, verbs, nouns, and personal pronouns etc., and make them as linguistic cues of stylistic (*St*) (see Table A1 in Appendix for detailed information).

#### Explanatory Variable 2: Synchrony Between Counselor and Counselee

The notion of synchrony is usually used to describe concurrent non-verbal behaviors (e.g., postures, gestures, facial expressions) that happened in the context of interpersonal communication (51, 52), referring to an interactive outcome that can only be achieved when participants share a common course of action/goal and constrain their behavior in a mutual relationship (53). We borrowed this terminology to refer to a synchronized conversation [cf. (54)] between the counselor and the counselee in SQA-OC, and examined it quantitatively through the following three aspects, i.e., topic consistency (TC), linguistic style matching (LSM), and emotional similarity (ES).

##### Topic Consistency Measurement

The research on topic consistency was to explore whether the counselors respond to multiple topics mentioned by the counselees in their question. Based on the word embedding based psychological topic detection method, we found seven topics related to psychological symptoms (i.e., depression and anxiety, suffering, social phobia, lack of interest, suicidal tendency, worried and afraid, and angry), and seven topics related to psychological factors (i.e., love, marriage, psychotherapy, work, interpersonal relationship, personal characteristic, and family). To accurately identify psychological topics, the distribution of high-frequency feature words in the text can be used (55). Therefore, we set up a seven-dimensional vector corresponding to seven topics to represent the topic diversity of psychological symptoms and influential linguistic cues, respectively. First, we matched the counselees' text with the words in psychological topic one by one, if a word under a topic appeared in a text, the corresponding element in the vector was changed to 1, otherwise, it was 0. Similarly, the responses corpus will perform the above operation. Thus, for each piece of SQA text, we get two topic vector representations of the counselees' questions and the corresponding response of counselors. Then, the Jaccard similarity was calculated to represent topic consistency between two texts and measured the shared attributes of sets A and B (where the set consists of 0 or 1). Jaccard similarity coefficient is a method for measuring the similarity of asymmetric binary attributes (56). The Jaccard similarity coefficient between counselees *i* and counselors *j* is: *J*(*R*_*i*_, *M*_*i*_) = |*R*_*i*_ ∩ *M*_*j*_||*R*_*i*_ ∪ *M*_*j*_|, where *R*_*i*_ is a topic vector representation of the counselee. *M*_*j*_ represents a topic vector representation of the counselor's response.|*R*_*i*_ ∩ *M*_*j*_| means the number of topics that co-occurred in both the counselee's question and counselor's response. |*R*_*i*_ ∪ *M*_*j*_| means the number of topics embedded in the text of the counselee's question and counselor's response.

##### Linguistic Style Matching Measurement

To operationalize the interactive and implicit aspects of the alliance in psychoanalytic psychotherapy, the language style matching metric is proposed, which is based on computerized text analyses performed using the software LIWC (37, 57). Rather than content-based aspects of language (e.g., using the counselee's description of feeling “livid” rather than “angry”), LSM represents the degree to which two people are producing similar rates of function words (e.g., pronouns, prepositions, and conjunctions) in their dialogue (57, 58). Indeed, the function word includes nine types: prepositions, auxiliary verbs, adverbs, conjunction, articles, quantifiers, negations, personal pronoun, and impersonal pronoun (37). Hence, we firstly use the CLIWC (Chinese Linguistic Inquiry and Word Count) program to calculate the proportion of function words in the text. Then, according to the method of LSM introduced by Ireland and Pennebaker (37), the LSM score of prepositions (preps) between texts from counselor and counselee is: *LSM*_*preps*_ = 1 − [(|*preps*_1_ − *preps*_2_|)/(*preps*_1_ + *preps*_2_ + 0.0001)], where *preps*_1_ represents the percentage of prepositions in the counselee's text, *preps*_2_ represents the percentage of prepositions in the counselor's text. The 0.0001 is added to the denominator to prevent an empty set, where the value of a function word category might be zero as a percentage of the entire text. This calculation is repeated for each of the nine function word categories. The nine category-level LSM scores are then averaged to yield a composite LSM score bounded by 0 and 1, where higher numbers represent greater LSM between counselee and counselor.

##### Emotional Similarity Measurement

Empathy is critical to a successful mental health support and is part of the therapeutic strategies in the training of counselors (59, 60). Empathy measurement has pre-dominantly occurred in synchronous, face-to-face settings (61, 62). It is unknown that such a computational approach to study empathy can be applied to an asynchronous, text-based context (63). Also, while previous NLP research has focused predominantly on empathy as reacting with emotions of warmth and compassion (64), or focusing on speech-based settings (61, 65), a separate but key aspect of empathy is to communicate a cognitive understanding of others (66). Given that millions of people use text-based platforms for mental health support, understanding empathy in SQA-OC has practical significance in this study.

In this study, we present a novel computational approach to understanding how empathy is expressed in SQA-OC. Empathy is a complex multi-dimensional construct with two broad aspects related to emotion and cognition (67). The emotional aspect relates to the emotional stimulation in reaction to the experiences and feelings expressed by a counselee. The cognitive aspect is a more deliberate process of understanding and interpreting the experiences and feelings of the counselees and communicating that understanding to them (60). Here, we study expressed empathy in text-based mental health support – empathy expressed or communicated by peer supporters in their textual interactions with seekers [cf. (68)].

Specifically, we used CLIWC to extract the emotion-related linguistic cues from texts of the counselee's question and the corresponding response of counselors, including seven dimensions: emotion, positive emotion, negative emotion, anxiety, anger, sadness, and love (see Table A2 for detailed information). We use the similarity between the counselees' and the counselors' emotion-related linguistic cues to quantify the emotional similarity to characterize the empathy. We apply cosine distance to measure the similarity between vectors of emotion (“emo”)-related linguistic cues of counselee *i* and counselor *j*, which is ***emo***_*counselor j*_ and ***emo***_*counselee i*_. We then calculated the cosine similarity as follows:


$$\begin{array}{l} Empay\left(counselor\;i,counselee\;j\right)=\cos\theta \\ =\frac{emo_{counselee\;i\;}\times emo_{counselor\;j}}{\sqrt{{\left(emo_{counselee\;i}\right)}^{2}\times {\left(emo_{counselor\;j}\right)}^{2}}} \end{array}$$

where the numerator represents the dot emotion-related linguistic cues vectors between counselee i and counselor j, the denominator represents the modular product of these two vectors.

### Explainable Machine Learning Method

Taking the perceived helpfulness of the public to the SQA-OC as the dependent variable, and linguistic cues from counselees' questions, counselors' responses, and their synchronous interaction between them as the independent variables, utilizing XML regressions, we proposed prediction models for perceived helpfulness of SQA-OC.

Specifically, we used linear machine learning regression like linear regression, ridge regression, Lasso regression, support vector regression (linear kernel), as well as non-linear machine learning regression like random forest, to build a prediction for perceived helpfulness of SQA-OC. We used mean absolute error (MAE) to evaluate the performance of different algorithms and feature sets in the model, used the ten-fold cross-validation to select the best predictive model. Shapley values are a widely used approach from cooperative game theory that come with desirable properties. Utilizing explainable artificial intelligence (XAI) method based on the Shapley values, we identified the influential features from all independent variables, as well as how they contribute to the perceived helpfulness of SQA-OC. SHAP values represent a feature's responsibility for a change in the model output (69). SHAP values offer two important benefits. First, global interpretability, namely the SHAP values can show how much each predictor contributes, either positively or negatively, to the target variable. Second, local interpretability, namely each observation gets its own set of SHAP values. Traditional variable importance algorithms only show the results across the entire population but not on each individual case, while the local interpretability enables us to pinpoint and contrast the impacts of the factors. SHAP value greatly increases the transparency of machine learning and has been implemented in many research and industry scenarios (70).

By accumulative the SHAP values of each feature, we quantified the positive and negative influence of different types of features on the perceived helpfulness. Let the amount of data is *M*. If the number of feature in feature set *F* is {1, 2, …, P}, the SHAP values of the these features are:


$$\left[\begin{array}{ccc} SHAP_{1,1} & \cdots & SHAP_{1,P}\\ \vdots & \ddots & \vdots \\ SHAP_{M,1} & \cdots & SHAP_{M,P} \end{array}\right],$$

Therefore, the positive SHAP value of the feature set *F* is: $SHAP_{F}^{+}=\overset{P}{\underset{i=1}{\sum }}\frac{\left(\overset{M}{\underset{i,j=1}{\sum }}SHAP_{i}^{j}\right)}{X_{i}}$, $SHAP_{i}^{j}>0,X_{i}\in \left[X_{1},X_{2},\ldots ,X_{P}\right],\;X_{i}\;$ is the amount of positive SHAP values for an specific feature *i*. We calculated the negative SHAP value in the same way. In addition, to classify features with different predicting power and influence, we calculated the relationship for SHAP values of each features using Pearson correlation coefficient.

**Figure 1:**
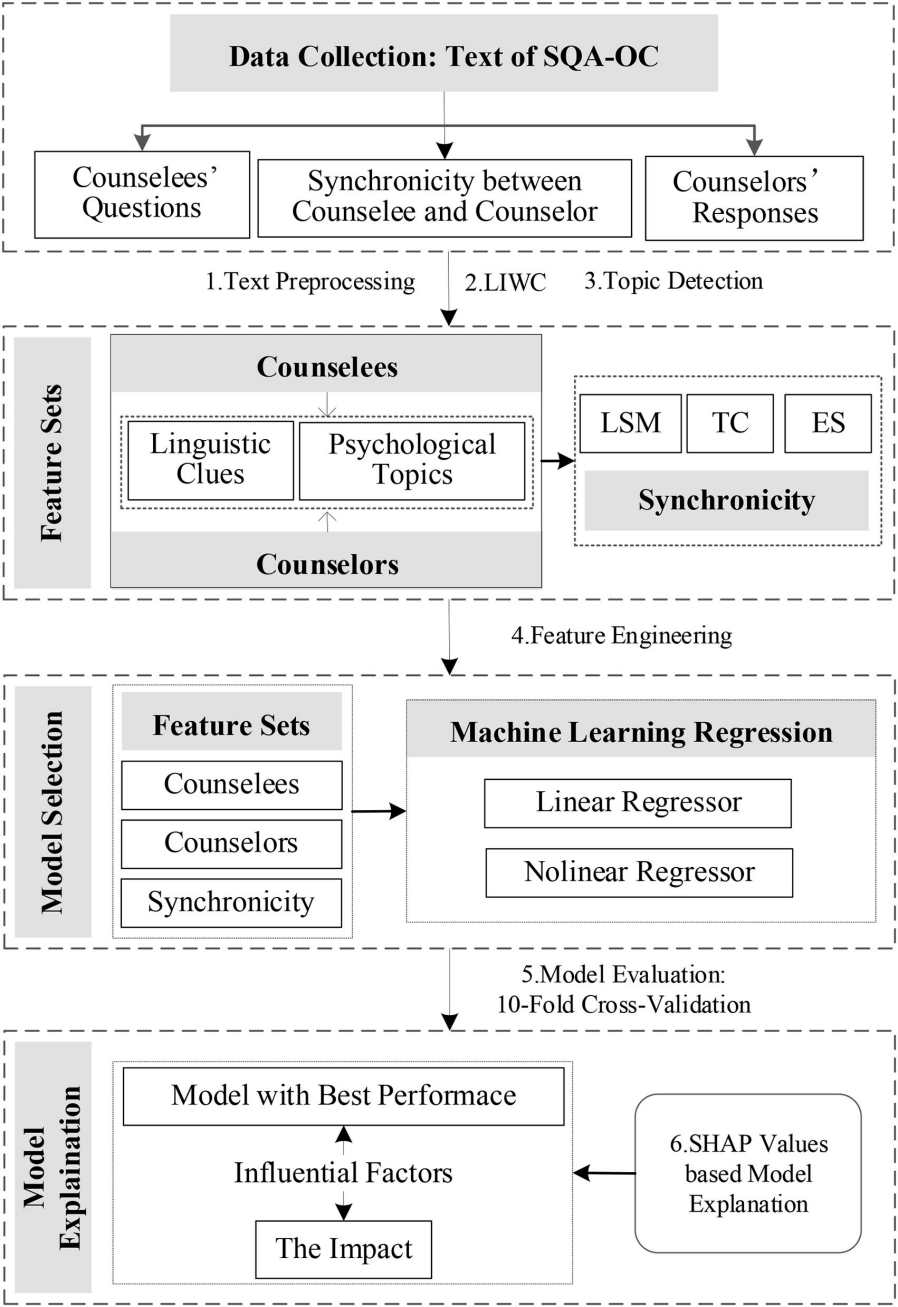
Research methods and processes.

The research methods and processes we proposed are shown in Figure 1.

## Results

### The Predictive Model of Perceived Helpfulness of SQA-OC

To build a predictive model of perceived helpfulness of SQA-OC with good performance and interpretability, we proposed feature sets of linguistic cues and specific combinations of their sources, including counselees' qusetions (i.e., “counselee” in Table 1), counselors' responses (i.e., “counselor” in Table 1), and the synchrony between counselor and counselee (i.e., counselee_counselor_sync in Table 1), and used machine learning regressors (i.e., linear regression, ridge, lasso, SVR and random forest in Table 1) to build the prediction. Further, we use MAE to evaluate the performance of different predictive models, and use the SHAP value to explain the model with the best performance.

**Table 1:** Performance of different algorithms and features sets in the model.

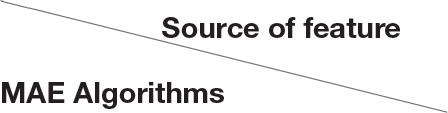	**Counselors**	**Counselees**	**Counselor-counselee synchrony**	**All three sources**	**Mean MAEs of Algorithms**
Linear regression	2.1715	2.2141	2.1625	2.0949	2.1488
Ridge	2.1726	2.2147	2.1627	2.0953	2.1493
Lasso	2.2598	2.2598	2.2598	2.2598	2.2598
SVR	2.1708	2.1977	2.1453	2.0812	2.1368
Random forest	1.8586	2.2640	2.1865	1.8555	2.0060
Mean MAE	2.1255	2.2301	2.1834	2.0773	

As shown in Table 1, we got the performance for predictive models with different algorithms and feature sets through the MAE values. We can see that the random forest based on the combination of features from counselees, counselors, and the synchrony between them achieved the lowest MAE among all predictions. Specifically, for predictions with a larger number of features, compared with other linear prediction algorithms, the non-linear random forest algorithm achieves a lower MAE. For for predictions with a small number of features, compared with the non-linear prediction algorithm (i.e., random forest), the support vector regressors with linear kernel achieves a lower MAE. In general, the fandom forest based on the combined feature sets of counselees, counselors, and their synchrony has achieved the best performance, and its MAPEs are 0.20108, 0.211445, and 0.228419, respectively. Non-linear random forest and linear support vector algorithms are better than other algorithms in the prediction.

In addition, we further selected the effective features that can improve the performance of the model from the feature set containing all variables of linguistic cues. Specifically, using the random forest and SHAP value-based XML method, we calculated the SHAP values for each of the feature in the variables, and ranked these features from the highest to the lowest based on the SHSP values. Then, we added each of the features to the random forest regressors according to the ranked order, and calculate the MAE value fro the regressors after adding new features each time. Finally, as shown in Table 1, we found that the top 52 most important features achieved the highest performance: 1.8556.

### The Influence of Linguistic Cues on the Perceived Helpfulness

#### The Influence of Linguistic Cues With Different Sources and Types on the Perceived Helpfulness

To further analyze the impact of different sources (i.e., counselors' question, counselees' response, synchrony between them) and types (i.e., *AP, SP, CP, PP, BP, Dr, TO, Rev, PC, IL, St, and CSS*) of linguistic cues on the predictive model of perceived helpfulness of SQA-OC, we calculated the cumulative SHAP values of the features mentioned above. The results are shown in Table 2.

**Table 2:** Cumulative SHAP values for different sources and types of linguistic cues in the perceived helpfulness predictive model.

	**Counselors source**			**Counselees source**			**Counselor-counselee synchrony**			
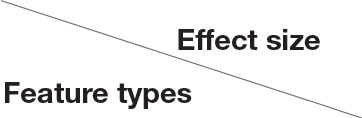	**Negative effect**	**Positive effect**	**Total effect**	**Negative effect**	**Positive effect**	**Total effects**	**Negative effect**	**Positive effect**	**Total effects**	**Total effects**
Affective processes	−0.1090	0.1082	0.2172	0	0	0	\	\	\	0.2172
Biological processes	−0.0775	0.0713	0.1488	0	0	0	\	\	\	0.1488
Cognitive processes	−0.5318	0.2821	0.8139	0	0	0	\	\	\	0.8139
Drives	−0.0592	0.0060	0.0653	0	0	0	\	\	\	0.0653
Informal language	−0.0942	0.1055	0.1998	−0.0047	0.0579	0.0625	\	\	\	0.2623
Numbers	−0.0576	0.0115	0.0691	0	0	0	\	\	\	0.0691
Perceptual processes	−0.0709	0.1291	0.2000	0	0	0	\	\	\	0.2000
Personal concerns	−0.1341	0.1781	0.3123	0	0	0	\	\	\	0.3123
Personal pronouns	−0.4995	0.7505	1.2500	0	0	0	\	\	\	1.2500
Prepositions	−0.0685	0.0118	0.0803	0	0	0	\	\	\	0.0803
Relativity	−0.0458	0	0.0458	0	0	0	\	\	\	0.0458
Social processes	−0.0409	0	0.0409	0	0	0	\	\	\	0.0409
Stylistic	−0.6109	1.2431	1.8539	0	0.1074	0.1074	\	\	\	1.9613
Time orientations	−0.1785	0.2092	0.3877	0	0	0	\	\	\	0.3877
AffectSIM	\	\	\	\	\	\	−0.0300	0.0636	0.0936	0.0936
LSM (mean)	\	\	\	\	\	\	−0.0255	0.0288	0.0544	0.0544
SymptomsSIM	\	\	\	\	\	−0.0279	0.0387	0.0665	0.0665	\

See Table A1 for definitions and descriptions of the types of language cues in the above table.

For the influence of different feature sources, first, we find that all the three sources improve the performance of the predictive model, and all these three sources have an incremental effect in improving the performance of the prediction. In terms of the relative differences, the features from counselors' response have the greatest impact on the perceived helpfulness, and its cumulative SHAP value is 6.093 (positive value is 3.2436 and a negative value is −2.8497), accounting for 93.38% of the total SHAP value; It is much higher than influence of the linguistic cues from counselees (the overall SHAP value is 0.1699, accounting for 2.60%) or the synchrony between counselees and counselors (the overall SHAP value is 0.2621, accounting for 4.02%).

For the influence of different types of features, we calculated and analyzed the cumulative SHAP values of different types of features in the predictive model. Among the counselor-sourced features set, stylistic, affective processes, biological processes, cognitive processes, drives, informal language, perceptual processes, personal concerns, personal pronouns, prepositions, relativity, social processes, stylistic, time orientations, were the influential types of linguistic cues in predicting the perceived helpfulness, accounting for 85.82% of the total effect. Among them, stylistic, cognitive processes, and personal pronouns were the top three most influential linguistic cues with SHAP values of 1.8539, 1.2500, and 0.8139, respectively, accounting for 60.04% of the total effect. In the counselee-sourced features set, informal language (SHAP value of 0.0625) and stylistic (SHAP value of 0.1074) features were the influential types of linguistic cues. Among the feature set of the synchrony between counselor and counselee, emotional similarity (0.0936) and topic consistency (0.0665) were the influential linguistic cue types.

In addition, for the way that different types of linguistic cues influence the perceived helpfulness, we analyzed the positive and negative influence of different types of linguistic cues on the perceived helpfulness, as shown in Table 2. The results show that for counselor-sourced linguistic cues, except for relativity, social processes, which only reduce perceived helpfulness, other types of linguistic cues may both enhance and reduce perceived helpfulness. For the different types of counselees- sourced language cues, stylistic only increases perceived helpfulness, while informal language may decrease and increase perceived helpfulness. For different types of linguistic cues from synchrony between counselor and counselee, emotional similarity, linguistic style matching, and topic consistency of symptom may both increase and decrease perceived helpfulness. We can see that counselors-sourced linguistic cues of relativity, social processes are risk factors of the perceived helpfulness, while counselees-sourced stylistic are facilitators.

#### The Way That Top-Ranked Linguistic Cues Influence the Perceived Helpfulness

**Figure 2:**
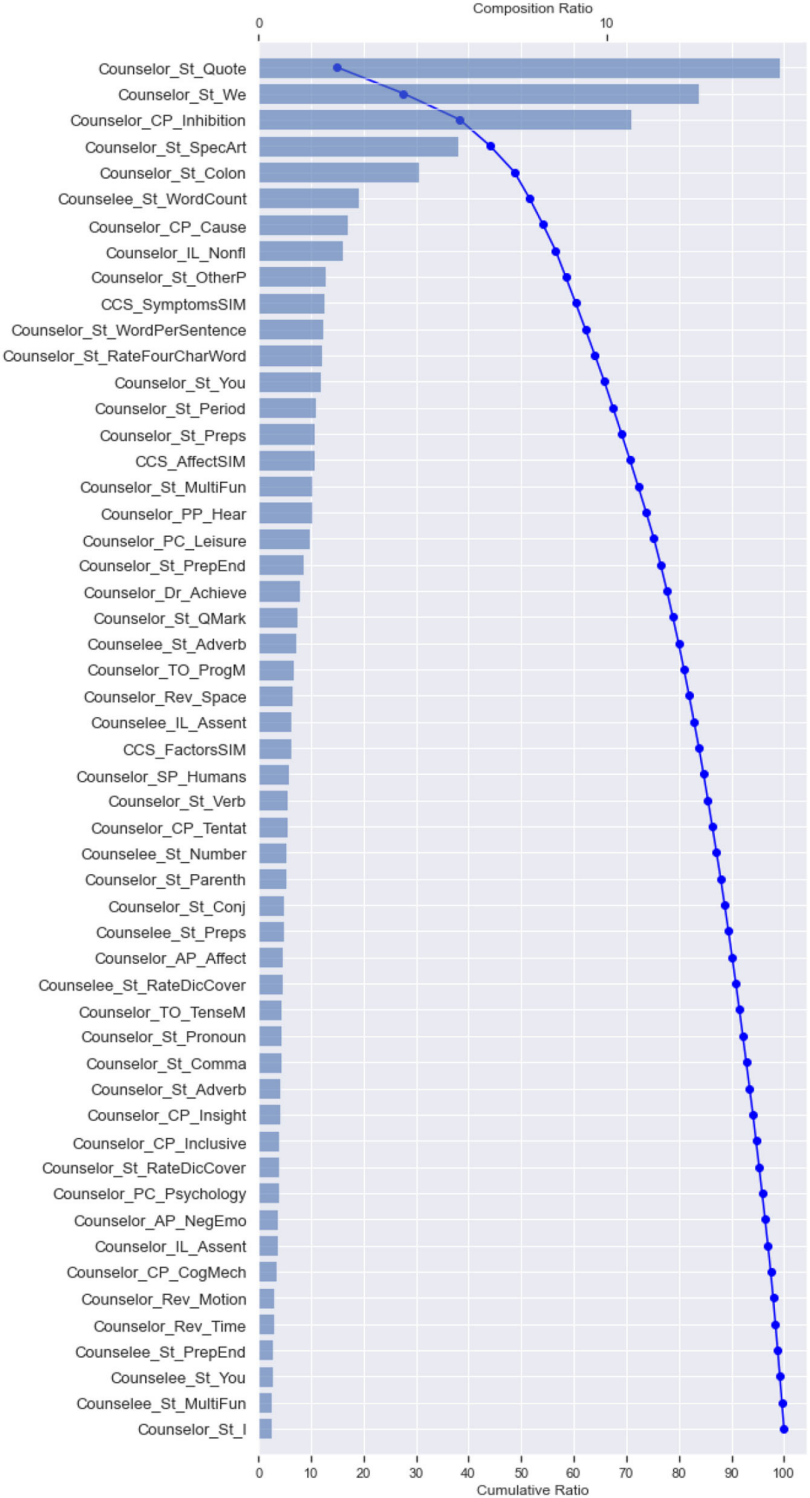
Cumulative SHAP values for top 40 most important linguistic cues in the perceived helpfulness predictive model (In this figure shows the top 40 tokens affecting perceived helpfulness, in order of importance, as determined by the SHAP summary output).

First, to obtain the most influential linguistic cues for the perceived helpfulness, we calculated and ranked the SHAP values of different features, as shown in Figure 2. For the cumulative SHAP values of the features in the predictive model, the top-33 most influential features contributed about 90% of the influence, and the top-40 influential features contributed more than 95% of the influence. Specifically, The top 33 features include (1) linguistics clues of counselee-sourced stylistic; (2) linguistics clues of counselor-sourced, i.e., stylistic, personal pronouns, cognitive processes, biological processes, cognitive processes, biological processes, personal concerns, affective processes, time orientations, perceptual processes; (3) linguistics clues of synchrony between counselor and counselee-sourced topic consistency of symptom.

**Figure 3:**
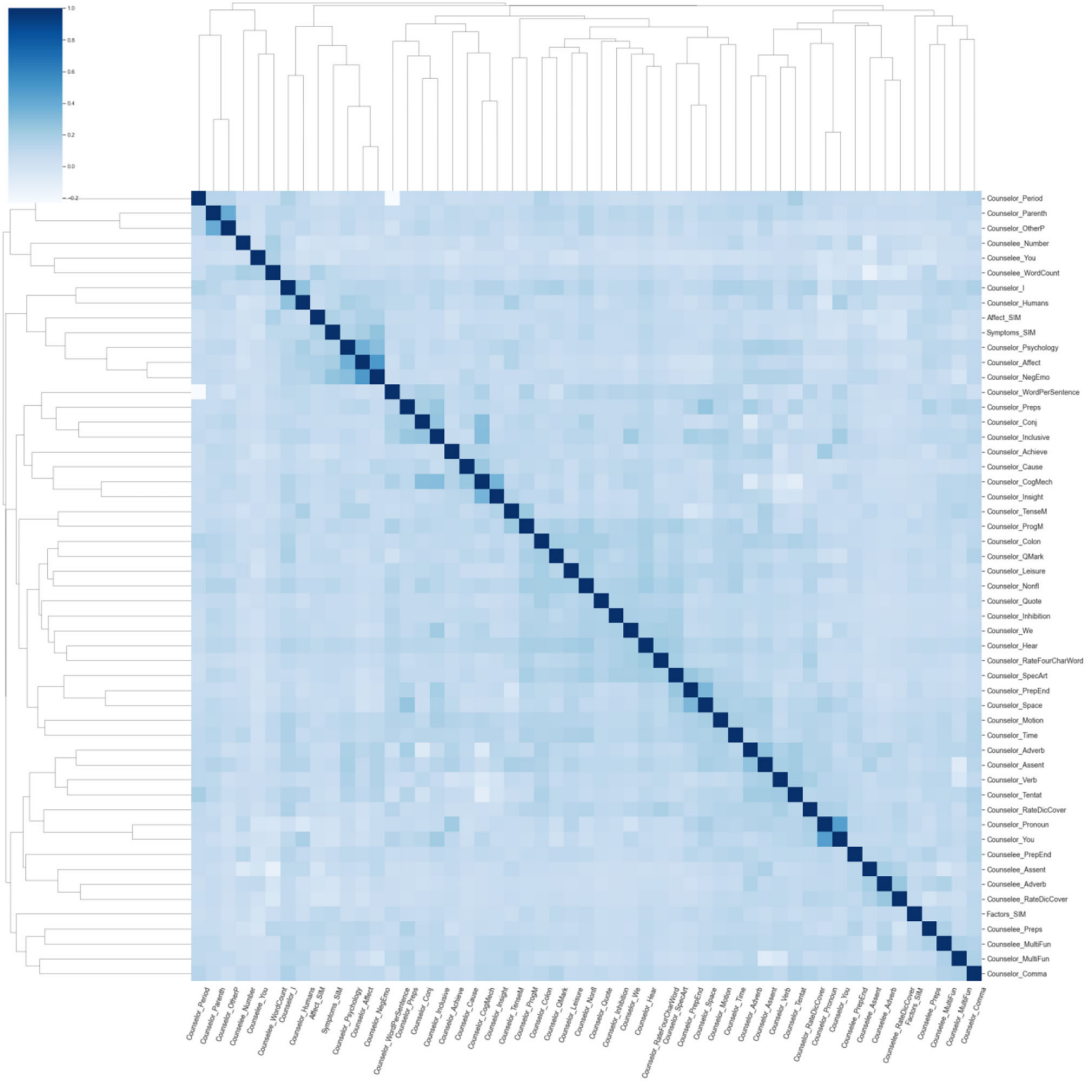
The clustering diagram based on SHAP value of the influential factors.

**Figure 4:**
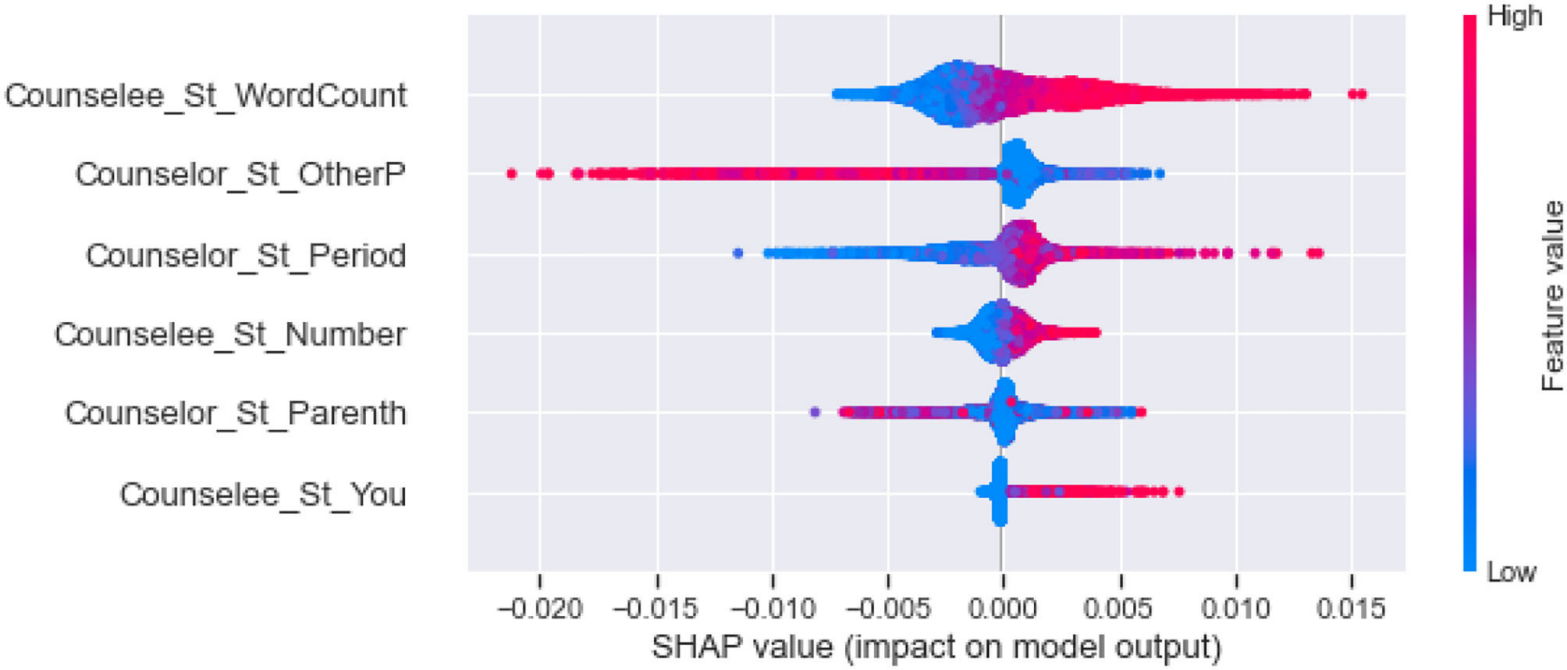
The SHAP summary plots about the adjustment to the predicted in perceive helpfulness numbers (x-axis) for each of the first type features.

**Figure 5:**
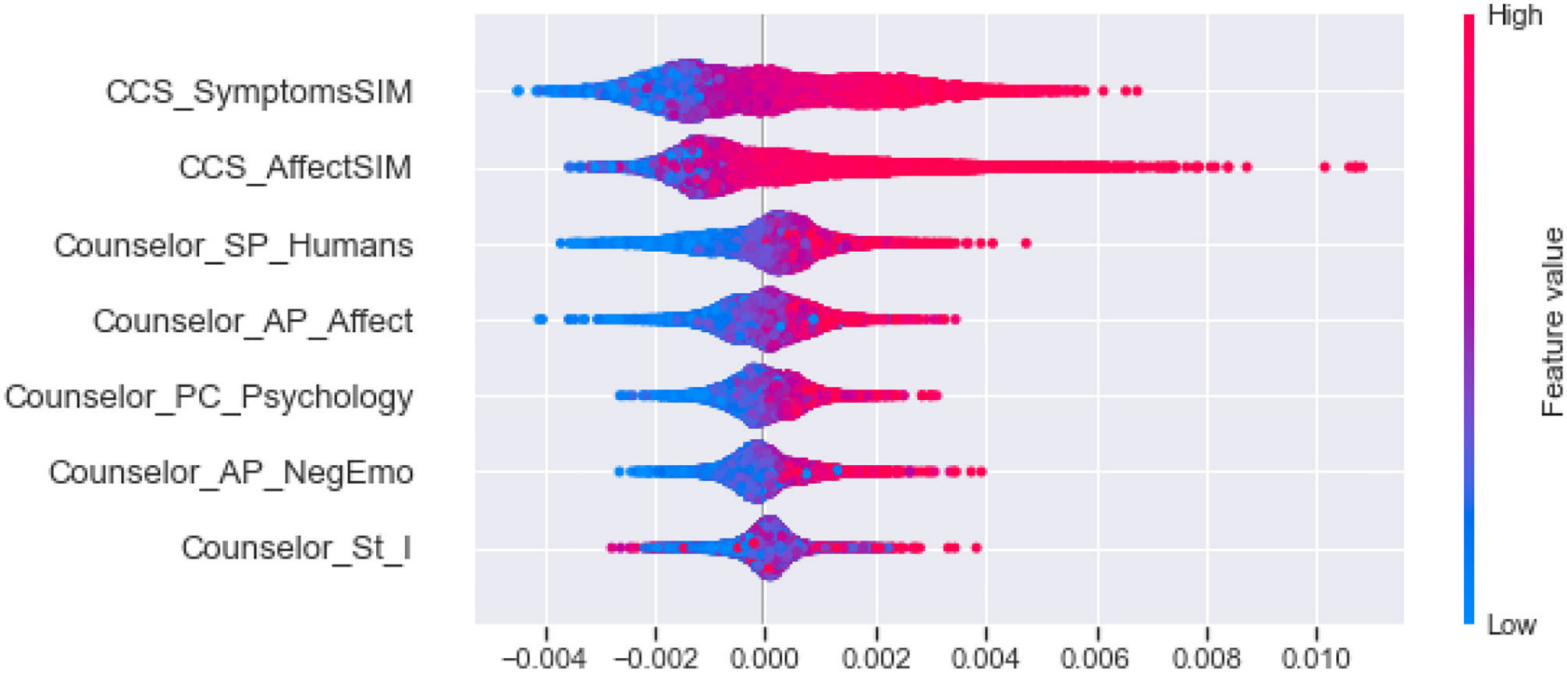
The SHAP summary plots about the adjustment to the predicted in perceive helpfulness numbers (x-axis) for each of the second type features.

**Figure 6:**
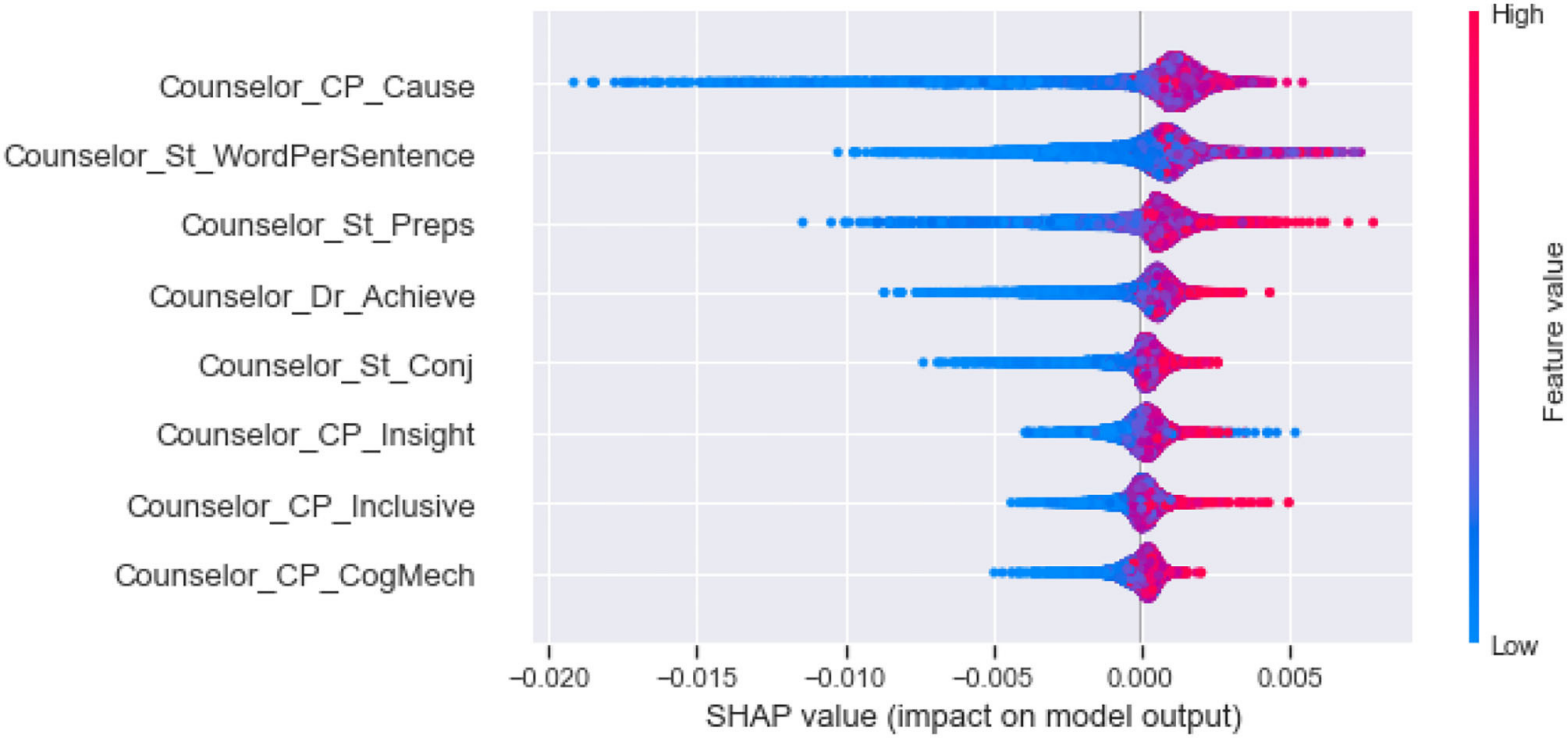
The SHAP summary plots about the adjustment to the predicted in perceive helpfulness numbers (x-axis) for each of the third type features.

**Figure 7:**
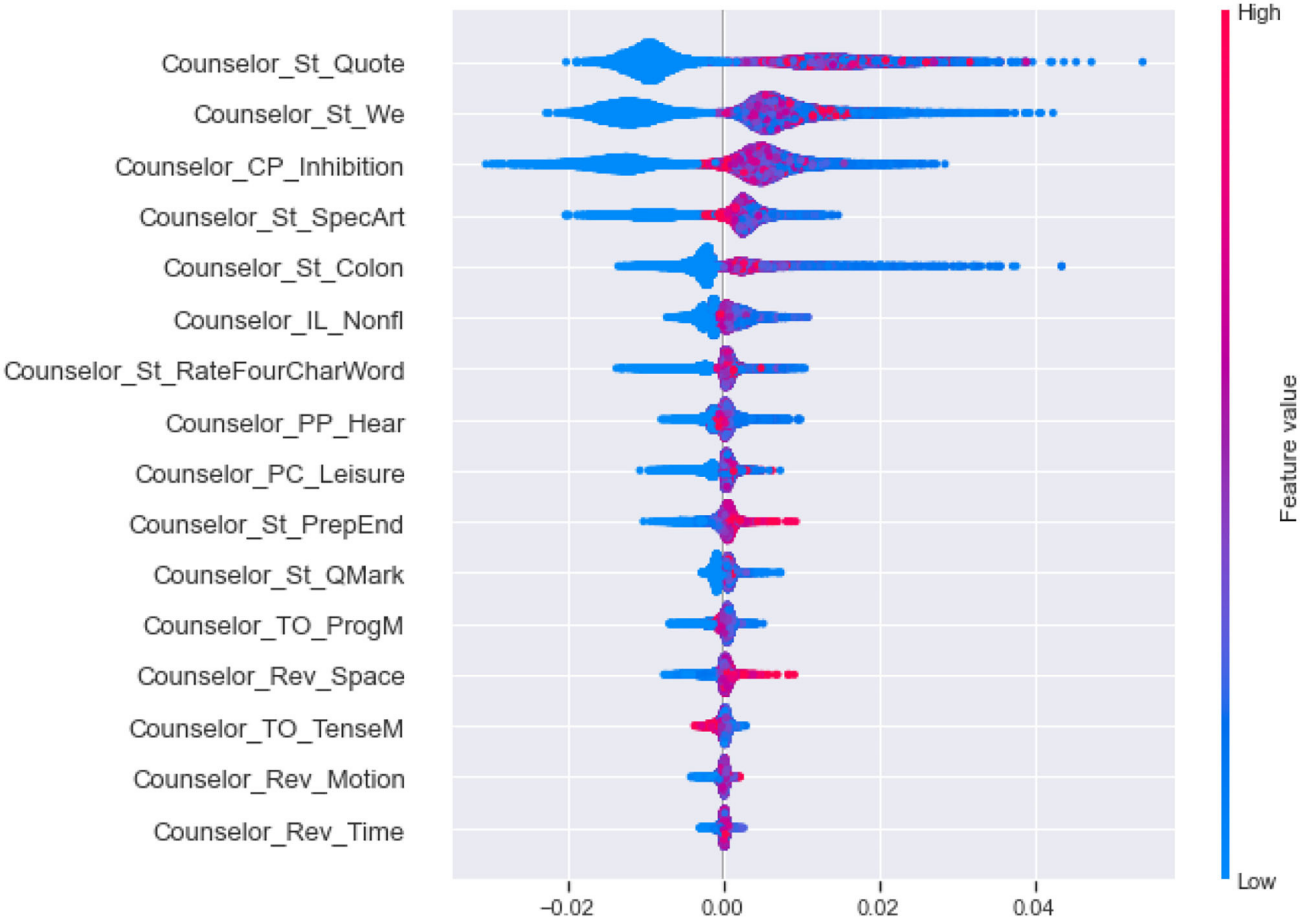
The SHAP summary plots about the adjustment to the predicted in perceived helpfulness numbers (x-axis) for each of the fourth type features.

**Figure 8:**
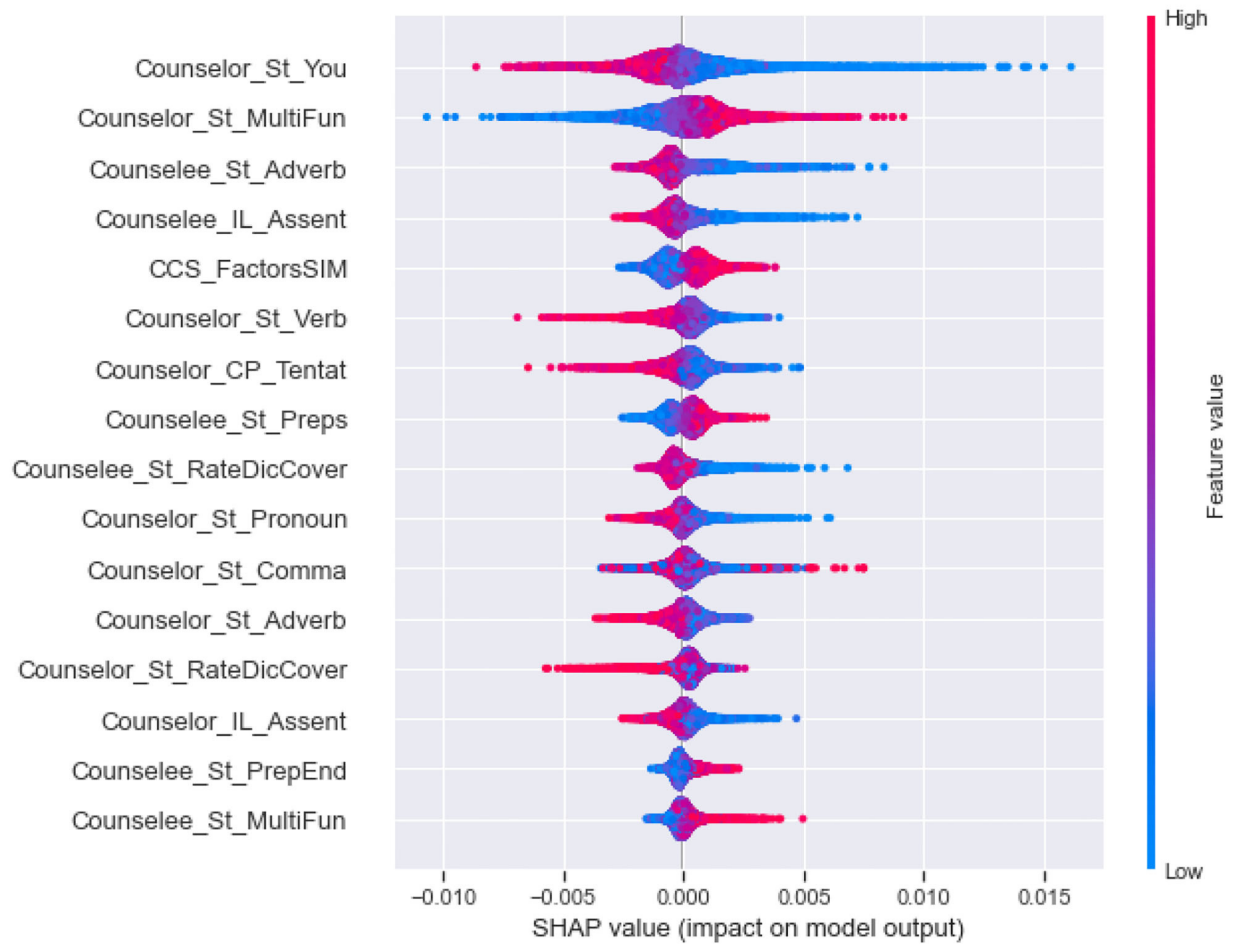
The SHAP summary plots about the adjustment to the predicted in perceive helpfulness numbers (x-axis) for each of the fifth type features.

Second, as shown in Figure 3, we clustering SHAP values of the effective linguistic cues, divided them into five categories, and analyze their influence in each category by the global interpretability of the model, as shown in Figures 4–**8**. In these figures, each plot is made up of thousands of individual points from the training data set such with a higher value being more red, and a lower value being more blue. This is depicted by the “feature value” bar on the right of each plot. Therefore, if the dots on one side of the central line are increasingly red or blue, that suggests that increasing values or decreasing values, respectively, move the predicated perceived helpfulness in that direction. Take Figure 4 as an example, lower word count values in stylistic from counselee (blue dots) are associated with a relatively lower perceived helpfulness. To briefly summarize, we detailed them into five typical patterns below according to the clustering results.

(1) As shown in Figure 4, the influential linguistic cues in the first category include linguistic cues of stylistic from counselees and counselors. Their influence on perceived usefulness can be divided to two types. For the first type, when values of the linguistic cues is at a high level, it improves the perceived usefulness; when its value is at a low level, it reduces the perceived usefulness. For example, *WordCount, You and Number* from counselees, *Period* from counselors belong to the first type. For the second type, when the value of the factors is at a high level, it decreases the perceived usefulness; When the value of the factors is at a low level, it improves the perceived usefulness, such as *Parenth and OtherP* from counselors.
(2) As shown in Figure 5, the second type of influential linguistic cues include stylistic, social processes, personal concerns, effective processes from counselors, and *AffectSIM, SymptomsSIM* from the synchrony between counselor and counselee. In addition to the linguistic cues of *I* from counselors, other linguistic cues show a consistent impact on the perceived usefulness, that is, when the value of the factor is at a high level, it improves the perceived usefulness, and when it is at a low level, it decreases the perceived usefulness.
(3) As shown in Figure 6, the third type of influential linguistic cues include stylistic, drive, cognitive processes from counselors, and show a consistent impact on the perceived usefulness, that is, when the value of the factor is at a high level, the perceived usefulness is improved, and when the value of the factor is at a low level, the perceived usefulness is reduced, for example, the stylistic (i.e., *WordPerSentence, Preps, Conj*), Drive (i.e., *Achieve*), cognitive processes (i.e., *Cause, CogMech, Insight, Inclusive*).
(4) As shown in Figure 7, the fourth type of influential linguistic cues include time orientations, stylistic, perceptual processes, informational language, cognitive processes, relativity, and personal concerns from counselors, and show a complex impact on the perceived usefulness. First, when the value of the factor is at a high level, it slightly improves the perceived usefulness; When its value is at low level, it significantly reduces or improves the perceived usefulness, such as language cues of stylistic (i.e., *Quote, We, SpecArt,Colon,RateFourCharWord*), cognitive processes (i.e., *Inhibition*), relativity (i.e., *Nonfl, Time*), perceptual processes (i.e., *Hear*), personal concerns (i.e., *Leisure*). Second, when the value of the linguistic cues is at a high level, it improves the perceived usefulness; When its value is at low level, it reduces the perceived usefulness, such as stylistic (i.e., *PrepEnd*), relativity (i.e., *Time, Motion*) from counselors. Third, when the value of the linguistic cues is at a high level, it reduces the perceived usefulness; When its value is at low level, it improves the perceived usefulness, such as language cues of time orientations (i.e., *TenseM*).
(5) As shown in Figure 8, the fifth type of influential linguistic cue includes stylistic, time orientations, informational language, cognitive processes, perceptual processes from counselors, stylistic and informal language from counselees, and linguistic cues from the synchrony between counselor and counsellee, and shows two effects on perceived usefulness. First, when the value of the linguistic cues is at a high level, it improves the perceived usefulness; When its value is at low level, it reduces the perceived usefulness, such as linguistic cues of stylistic (i.e., *MultiFun, PrepEnd*) from counselors, linguistic cues of stylistic (i.e., *Preps, MultiFun*) from counselees, and linguistic cues from the synchrony between counselor and counselee (i.e., *Factors SIM*). Second, when the value of the linguistic cues is at a high level, it reduces the perceived usefulness; When its value is at low level, it improves the perceived usefulness, such as language clues of stylistic (i.e., *You, Adverb,Verb, Pronoun*), cognitive processes (i.e., *Tentat*) and information language (i.e., *Assent*) from counselor, information language (i.e., *Assent, Adverb*) from counselees.

## Discussion

This exploratory research investigated automatic predictive methods and linguistic cues of the perceived helpfulness of SQA-OC. It puts forward prediction algorithm and factors with advantages, then discovers relevant influential factors from the interpretability. The findings of this study can be summarized in three parts: (1) algorithms and linguistic cues with advantages in predicting the perceived helpfulness; (2) the importance of the influential linguistic cues with different sources and types to the perceived helpfulness; (3) and the influence of these linguistic cues on the perceived helpfulness. We explained each part below and summarized the main contributions of this study.

### The Predictive Model

In terms of the predictive model on the perceived helpfulness of SQA-OC, this study found that the random forest algorithm combining a set of counselees' features, counselors' features, and counselor-counselee interactions achieved the best predictive performance and has potential practical application significance. Comparing with different predictive algorithms, our findings showed that the non-linear regression model performs better than the linear model, which is in line with previous studies of automatically predicting the perceived helpfulness of online service (22, 71, 72). More specifically, this study showed that the linear model achieves better performance with a smaller number of features, while the non-linear models can represent the non-linear relationship between features and are more suitable to use in predictive situations of high complexity.

In terms of the different sources of feature sets, the combination of features about counselees' questions, counselors' responses, and the synchrony between counselees and counselors achieve the optimal performance. One possible explanation is that the number of influential features in the counselee-source and counselor-counselee-source in the predictive model only contains two or four features, while the number of counselor-source contains 46. It has been suggested that the number of features positively predicts the complexity of the predictive model (73), which explains why the counselor-source is indispensable in the complicated predictive model owing to a large number of highly influential features.

Furthermore, we found that the linguistic cues from counselors are the most important, despite all three having incremental effects to improve the performance of the prediction. This is in line with previous findings which showed that the linguistic features of counselors, counselees, and counselor-counselee interactions can predict the therapeutic outcome (62, 74, 75). For the perceived helpfulness and therapeutic outcome of online counseling in the asynchronous and one-off service environment, these findings also suggested that the strategies and skills of counselors play a major role.

### The Importance of the Influential Linguistic Cues With Different Sources and Types to the Perceived Helpfulness

The interpretability gives machine learning the ability to explain or present their behaviors in understandable terms to humans (76), which is an effective tool to understand and improve the perceived helpfulness.

First, the global interpretability of the model identified and clarified the influential linguistic cues related to the perceived helpfulness, as well as their relative importance. The influential linguistic cues implied the influence of counselees' and counselors' attentional focus, thought processes, emotional states, and social relationships on the perceived helpfulness of SQA-OC. Specifically, for the counselor-source linguistic cues, the global interpretability of the model indicated stylistic, personal pronouns, cognitive processes, time orientations, personal concerns, affective processes, perceptual processes, informal language, biological processes, prepositions, numbers, drives, relativity, multifunction, social processes are the top-ranked predictors of perceived usefulness. These linguistic cues are widely believed to be related to individual psychological processes, such as cognitive process, emotion, and social relations (77). We provided evidence for the previous findings of counselors' linguistic cues and therapeutic outcomes in the online, asynchronous, and one-off service environment, and promoted the influencing factors of perceived usefulness in the SQA-OC context. For counselee-source linguistic cues, stylistic (i.e., length of texts, second person pronouns, numbers) and informal language are the influential factors. These linguistic cues usually explain who dominates a conversation and how they engage in the conversation, and predict the quality of relationships (77). For the linguistic cues of counselor-counselee synchrony sources, emotional similarity, topic (symptom) consistency, and language style similarity are the determinate predictors for perceived helpfulness. These findings are in line with researches of both perceived helpfulness (24, 38) and mainstream Therapeutic Change Process Research (TCPR) using computerized-text analysis (78, 79). In general, the global interpretability of the prediction provides insights into what makes a good SQA-OC and offers policy suggestions for the counseling platform to undertake professional training strategies for counselors.

**Figure 9:**
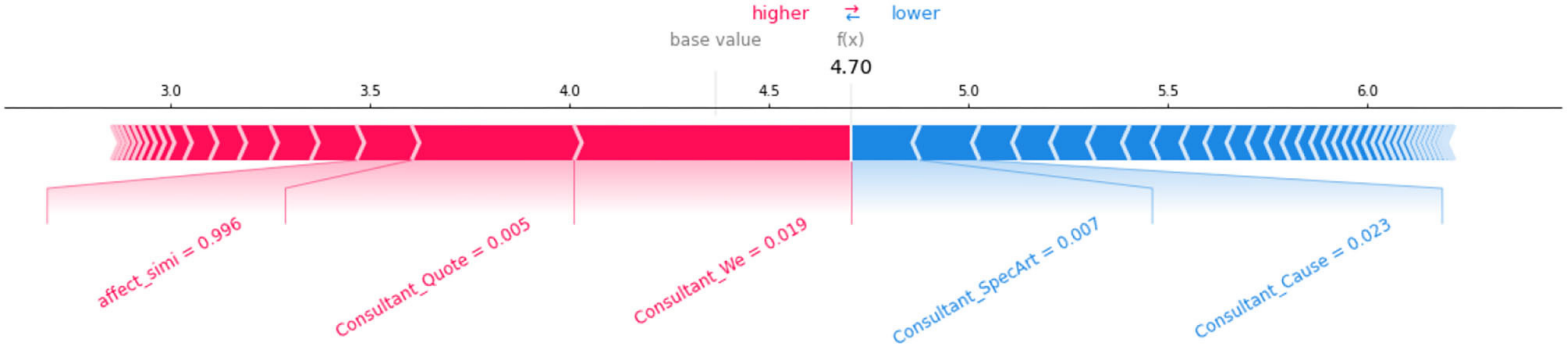
SHAP force plots supporting the local interpretability with a specific prediction.

Second, the local interpretability of this method produces both prediction and explanation for each response from counselors. As shown in Figure 9, for the prediction and explanation of an unvoted response we predicted (4.70 votes), we can see that the emotional similarity was 0.996, which ranked third among the positive factors, and the linguistic cues of *SpecArt* and *Cause* are top-ranked negative factors. Local interpretability of the prediction allows counselors to evaluate and improve their service in advance and facilitates the use of targeted counseling strategies.

### Influence of the Influential Linguistic Cues on the Perceived Helpfulness

This study examined the influence of influential factors on the perceived helpfulness of SQA-OC and summarized them into five styles of linguistic cues that can improve the perceived helpfulness of SQA-OC, namely “talkative”, “empathy”, “thoughtful”, “concise with distance”, and “friendliness and confident”.

The first pattern is characterized by a “talkative” style, with a high level of stylistic (*WordCount, You and Number)* from counselees, and linguistic cues of stylistic (*Period)* from counselors, which reflects attentional allocation and engagement of counselors and counselees in SQA-OC. It is consistent with previous research, indicating that a greater word count means people who are more dominant and engaged in the conversation. However, it is inconsistent with the finding of the use of second-person words, which is more important in predicting lower-quality relationships (37). The distinct use of stylistic linguistic cues by counselors and counselees compared to traditional TCPR, possibly because of the single QA format and text-based feature of SQA-OC. This format facilitates both counselees and counselors to provide help-seeking information at one time as much as possible, in which context counselors want to circumvent the ambiguity of response (32) or to highlight their authorship differing from other counselors' responses (80).

The second pattern is characterized by an “empathy” style, which reflects the emotional state and social relationships of counselors and counselees in SQA-OC. This style contains stylistic (i.e., *I*), social processes (i.e., *Humans*), personal concerns (i.e., *Psychology*), affective processes (i.e., *Affect, NegEmo*) from counselors, and the synchrony between counselor and counselee (i.e., *AffectSIM, SymptomsSIM*). People who are experiencing physical or emotional pain tend to have their attention drawn to themselves and subsequently use more first-person singular pronouns (Tausczik and Pennebaker, 2010). In line with previous studies using the computerized approach to study counseling progress, a high level of first-person pronouns, emotional words, along with similarity in affect and language style (35), are important factors in the higher-quality relationship between counselor and counselee. Emotional similarity reflects the emotional aspect of empathy which predicts a counselor's competency and conversation skills (34, 81), while LSM and topic consistency (i.e., *symptomSIM*) represent the cognitive dimension of empathy (54) and reflect unconscious inter-personal communication behavior that promotes mutual understanding and increases intimacy between the two parties (77). These three dimensions influence the counselees' perception of the overall counseling experience in SQA-OC. Therefore, the use of first-person singular pronouns and the more similar the counselor's response text is to the counselee's language style, emotional disposition, and symptoms, the more likely the counselees are to vote the SQA-OC experience as useful and helpful.

The third pattern is characterized by a “thoughtful” style, with mainly linguistic cues of stylistic, drive, and cognitive processes from counselors, which reflects counselors' thinking styles and intentions in SQA-OC. Counselors with this style use more words of cognitive process (i.e., *Cause, CogMech, Insight, Inclusive*) and prepositions, which implies they make more efforts in analyzing the symptoms and causes of counselees' psychological distress, proposing treatment strategies, and promoting their implementation, so as to relieve counselees' psychological distress. Linguistic words of the cognitive process like exclusion words and conjunctions capture people's cognitive complexity (77). By using the “thoughtful” style, counselors could create causal explanations to organize their thoughts in counseling.

The fourth pattern is characterized by a “concise with distance” style, which reflects engagement, cognitive load, and psychological distance between counselors and counselees in SQA-OC. This style contains linguistic cues of stylistic, first-person plural, cognitive processes, relativity, perceptual processes, and personal concerns from counselors. Counselors' response with high perceived usefulness tend to use a moderate number of words (i.e., *Quote, SpecArt, Colon, RateFourCharWord*) and first-person plural (i.e., *We*), cognitive processes (i.e., *Inhibition*), relativity (i.e., *Nonfl, Time*), perceptual processes (i.e., *Hear*), personal concerns (i.e., *Leisure*), as many prepositions as possible (i.e., *PrepEnd*), relativity (i.e., *Time, Motion*), fewer words of time orientations (*TenseM*). On the one hand, these findings somehow contrast with the fact that good speakers are more biased toward group focus [plural pronouns “we”, (82)], which shows group cohesion (37). On the other hand, research indicated that with high load in conversation, people speak more and used longer sentences, used more words, and more plural personal pronouns (83). Likewise, during high-quality counseling, counselors achieve a more balanced exchange of words with counselees as the conversation progresses (62). These findings may also confirm that a reasonable length of response and an appropriate expression of first-person, cognitive processes, perceptual processes, and relativity are the strategies for counselors in SQA-OC. In addition, for the use of words such as biological and perceptual process, and time orientations, a previous study suggested that there are no significant differences between good and poor counselors (33). This is inconsistent with the results of the present study, which may be due to the fact that the SQA-OC uses a single QA format instead of stepwise multiple counseling progress, leading counselors to use as many cognitive/ sensory/affective descriptions as possible to meet counselee's information demand and relieve his psychological distress.

The fifth pattern is characterized by a “friendliness and confident” style, which reflects the formality and thinking style of counselors and counselees in SQA-OC. As mentioned earlier, experienced counselors and counselees with this style use more functional words and conjunctions (i.e., *MultiFun, PrepEnd*), providing more complex and, often, concrete information about a topic. In particular, experienced counselors and counselees tend to use fewer second-person pronouns (i.e., *You*), verbs, cognitive processes (i.e., *Tentat*), and informal language (i.e., *Assent*), while more similar in analyzing the influencing factors of psychological distress (i.e., *Factors SIM*). Specifically, owing to less informal language (i.e., Assent, Adverb) improving perceived helpfulness, this finding complements the previous NLP approach which studies counseling conversations from the perspective of the counselees' linguistic features, namely that the influence of counselees' language output is equally important as the counselors to the counseling process. For these counselees, using more informal language may reduce the perceived helpfulness of SQA-OC. Furthermore, when people are uncertain or insecure about their topic, they use tentative language (37). Therefore, successful counselors are better at handling ambiguity in the conversation, and using more words is one of the effective strategies to make the conversation less uncertain and more concrete (32).

### Contribution and Limitation

The first contribution of this study is the application of the notion of perceived helpfulness in measuring the public's perception of SQA-OC. Although this notion has been applied in the field of online counseling research (3, 84), it is not always quantifiable and easily accessible in the online context. In addition, studies that use text mining technology to automatically measure, model, and predict the helpfulness of online counseling are still rare. In terms of to which extent the counselor's response is helpful (i.e., the social aspect) and the ranking/popularity of the counselor on the platform (i.e., the economic aspect), investigating the degree of online helpfulness votes of counselor's response maybe helpful to understand both the social impact and economic impact of online counseling. With the aid of text mining technology, we found that linguistic features of the counselor's responses and counselee's questions play a decisive role in the predictive model. Compared to the studies of shopping websites (i.e., TripAdvisor, etc.), whose helpful votes will be transformed into the direct economic benefits of the platform. The helpful votes of SQA-OC will be transformed into a more powerful, comfortable, and secure psychological resource for the counselees. Furthermore, we found the synchrony between counselor and counselee in the SQA-OC context, including the aspects of language style matching and topic consistency, but more attention has been paid to emotional and symptom similarity compared to the studies of other online service platforms. Previous scholars have expressed their concerns about the capacity and professionality of online counselors and argued that the service quality of online counseling is hard to guarantee (5). The results of this study showed that the public's perception of helpfulness can only be improved if it is synchronized and resonated with the counselees at both the semantic, cognitive, and emotional levels. Online counselors may improve their professional competence from the above points.

The second contribution is that we examined the online therapeutic relationship and developed a novel method to computerize the counselor-counselee interaction from the aspects of LSS, emotional similarity, and topic consistency. Previous studies have suggested that counselees may unveil at a faster rate when communicating with counselors online (85, 86) and get straight to the point rather than peacefully easing into a problem, due to the “disinhibiting effect” (84). However, little is known about how to measure the characteristics of text-based communication and the interaction pattern between counselor and counselee. Our study indicated five styles (“talkative”,“empathy”, “thoughtful”, “concise with distance”, and “friendliness and confident”) that may happen in the online therapeutic relationship, using large-scale discourse analysis. However, it is noteworthy that counselors may adopt more than one style in the actual practice settings.

We acknowledge that the working alliance is considered to be one of the most crucial factors in the counseling process (87, 88). Although we did not directly measure working alliance as a dependent variable, the measure of perceived helpfulness may be an indicator and reflection of working alliance. More specifically, the five styles lead to either higher or lower levels of perceived helpfulness in the counseling process, which is in line with the “repair-rupture” process of working alliance (89–91). This finding implies that online counselors should be more aware of their verbal responses in the first time of counseling process and adjust their future communication style to maintain the working alliance with the counselees.

This study has several limitations. Firstly, there was no demographic data such as age, gender, or occupation-level data in the estimation model due to the anonymity of the SQA-OC platform. Therefore, it is hard to identify to what extent the factor of age, gender, and occupations interact with the linguistic features of counselees and counselors. Although most counselees do not expose their personal information on the platform explicitly, their demographic information can be predicted from SQA behavior by utilizing advanced machine learning technology (92). Secondly, the computerized text-based analysis in this study has several limitations, such as the absence of non-verbal cues (93), issues of inhibition, and temporal fluidity (94). While this may adversely influence the strategies used in traditional counseling, web-based communication still has several strengths including stronger emotional disclosure (95) and a higher level of client empowerment (96). Future analysis can be improved by incorporating self-report or interview data of the counselees, to verify the perceived helpfulness of SQA-OC from a personal level.

In terms of physical distancing, web-based psychological services can effectively address crisis-related issues. While SQA-OC works as an effective and convenient pre-consultation service, its service quality can be further improved by integrating with the internet cognitive-behavior therapy (ICBT) services (97, 98). The integration may result in better treatment outcomes and wider usage of online mental health services for someone whose treatment outcome is not ideal in the traditional face-to-face modality or has the limitations of stigma, cost, or transportation (99).

## Conclusions

We presented a large-scale quantitative study on the online and asynchronous conversation between psychological counselees (i.e., counselees) and counselors on the SQA-OC platform. We proposed an interpretative predictive model to automatically measure the perceived helpfulness of SQA-OC and investigated the impact of linguistic cues in the three sources (counselor, counselee, counselor-counselee synchrony) on the model performance. We hope that this work can inspire the future improvement of online counseling platforms as well as the online counselors, for instance using actionable conversation strategies to improve the public's perception of the helpfulness of online counseling services.

## Data Availability Statement

Publicly available datasets were analyzed in this study. This data can be found here: https://www.xinli001.com/qa?source=pc-home.

## Author Contributions

YH, HL, and SL: conceptualization and writing—original draft preparation. YH and SL: methodology. HL and YH: formal analysis. HL, YH, SL, ZZ, and WW: writing—review and editing. HL: visualization. WW, ZZ, and YH: funding acquisition. All authors have read and agreed to the published version of the manuscript.

## Footnotes

^1^Available online at: https://www.xinli001.com/ (accessed April 20, 2022).

^2^ https://www.bazhuayu.com/

^3^Available online at: https://www.Jiandanxinli.Com/Public/2020/ (accessed April 12, 2022).

^4^ https://www.xinli001.com/qa/ask

^5^ https://github.com/fxsjy/jieba

^6^Available online at: https://radimrehurek.com/gensim/models/word2vec.html (accessed August 1, 2021).

^7^One Psychology platform categories the Q&A section in five blocks—Recommended answers (with questions), Recommended questions (with answers), Newest questions, Fine selection in last 30 days, and Offer a reward. Online counselees may browse the first four blocks to get help, and counselors may get paid in the last block if they answer some of the questions raised by the counselees.
