# Supplementary material for: Effective Prediction and Important Counseling Experience for Perceived Helpfulness of Social Question and Answering-Based Online Counseling: An Explainable Machine Learning Model
Source: Front Public Health. 2022 Dec 22;10:817570. doi: 10.3389/fpubh.2022.817570 (PMC9815621; doi:10.3389/fpubh.2022.817570)
Supplement: Supplementary file 1 [file Table_1.DOCX]

# Appendix

TableA1: Type and dimensions of the Linguistic cues

| Name of the subclass to which the variable belongs | Name of the variable | Abbreviation of variable name | Example |
| --- | --- | --- | --- |
| Affective processes (AP) | Emotional process words | Affect | Anger, gratitude, disappointment |
|  | Positive emotion words | PosEmo | Confidence, satisfaction, blessing |
|  | Negative emotion words | NegEmo | Worry, suspicion, revenge |
|  | Anxiety words | Anx | Uneasiness, struggle, tension |
|  | Angry words | Anger | Damn, complain, destroy |
|  | Sad words | Sad | Heartache, depression, physics |
| Social processes (SP) | Social process words | Social | Understanding, choosing, questioning |
|  | Family words | Family | In laws, brothers, granddaughters |
|  | Friend words | Friend | Companions, friends, comrades |
| Cognitive processes (CP) | Insight words | Insight | Understand, suddenly realize, experience |
|  | Causal word | Cause | Cause, let, become |
|  | Gap Words | Discrep | Insufficient, expected, should |
|  | Provisional words | Tentat | About, come on, almost |
|  | Exact word | Certain | No doubt, necessity, guarantee |
| Perceptual processes (PP) | Perceptual process words | Percept | Warmth, experience, gaze |
|  | Visual words | See | Appearance, shiny, green |
|  | Auditory words | Hear | Shout, hear, talk |
|  | Sensory word | Feel | Smooth, elastic, touch |
| Biological processes (BP) | Physiological process words | Bio | Dizziness, hugging, sweating |
|  | Body words | Body | Neck, skin, stomach |
|  | Health words | Health | Insomnia, doctor, exercise |
|  | Sex words | Sexual | Sex, sex, nudity |
|  | Feeding words | Ingest | Digest, eat and cook |
| Drives (Dr) | Achievement words | Achieve | Good at, responsible, expert |
| Time orientations (TO) | Past tense | PastM | went, ran, had |
|  | Present tense | PresentM | Is, does,hear |
|  | Future tense | FutureM | Will, gonna |
|  | Progressive tense | ProgM | had, done, gone |
|  | Tense marker | TenseM | Already, before, in the future |
| Relativity (Rev) | Relative word | Relative | Before, compared to, reach |
|  | Mobile word | Motion | Pass, participate, approach |
|  | Spatial words | Space | Inside, street, stage |
|  | Time word | Time | Period, past, autumn |
| Personal concerns (PC) | Working words | Work | Factory, interview, salary |
|  | Leisure words | Leisure | Singing, relaxing, vacation |
|  | Family words | Home | House, family, pets |
|  | Money words | Money | Rich, annual salary, discount |
|  | Religious words | Religion | God, mercy, faith |
|  | Death words | Death | Death, suicide, will |
| Informal language (IL) | Should and word | Assent | Yes, really, good |
|  | Pause filler | Nonfl | Well, then, that |
|  | Filler filler | Filler | Just, like, say |
|  | obscene language | Swear | Fuck you, retarded, idiot |
| Stylistic (St) | Articles | Article | A, an, |
|  | Prepositions | Preps | To, with, above |
|  | Auxiliary verbs | AuxVerb | Am, will, have |
|  | Common Adverbs | Adverb | Very, really, quickly |
|  | Conjunctions | Conj | And, but, whereas |
|  | Negations | Negate | No, not, never |
|  | Common verbs | Verb | walk, went, see |
|  | Numbers | Number | Second, thousand |
|  | Quantifiers | Quant | Few, many, much |
| Synchrony between counselor and counselee | emotional similarity | AffectSIM | \ |
|  | Similarity in Using Prepositions | PrepsSIM | \ |
|  | Language Style Matching | LSM | \ |
|  | Similarity in Expressing psychological Symphoms | SymptomsSIM | \ |
|  | Similarity in Expressing psychological Factors | FactorsSIM | \ |

Table A2: The topics of psychological problems related to the SQA-OC

| Type of Topics | Topics | High-frequency word (Ranking in order) |
| --- | --- | --- |
| psychological problems | Depression and anxiety | depression, anxiety, insomnia, obsessive-compulsive disorder, depressive symptoms, diagnosis, bipolar, despair, violence, shadows, trauma, extreme, headaches, waking up, staying up, dreaming |
|  | Suffering | unhappy, sad, uncomfortable, wronged, embarrassed |
|  | Social phobia | communication, self-abasement, introversion, sensitivity, lack of self-confidence, dissocial, cowardice, dependence, eye contact, avoidance, conversation |
|  | Lack of interest | no interest, no drive, no confidence, no enthusiasm, no desire |
|  | Suicidal tendency | suicide, self-harm, tendency, breakdown, fear of pain, despair, escape, regret, torture, bad |
|  | Worried, afraid | fear, worry, tension, doubt, struggle, avoidance, rejection, nausea |
|  | Angry | anger, dislike, tantrums, bullying, grievance, disgust, blame, rejection, excess, ugliness, grumpiness, dissatisfaction, selfishness, trust, respect |
| influential factors | Love | love, boyfriend, relationship, girlfriend, heterosexual, confession, break up, good feeling, gay, single, Ex, meet, ex-boyfriend, reunion, first love, ex-girlfriend, Cold War, entanglement, long-distance relationship |
|  | Marriage | marriage, divorce, children, pregnancy, wife, man, mother-in-law, husband, married, sex, birth, in-laws |
|  | Psychotherapy | treatment, diagnosis, pandemic, anxiety, disorder, medication, mental illness, withdrawal, bipolar, character, cognition, character disorder, schizophrenia |
|  | Work | job, graduation, resignation, income, economy, pressure, development, unemployment, job-hopping, career, boss |
|  | Interpersonal  relationship | communication, character, contact, friend, speech, relationship, conversation, eye contact, dealing, indifference, impression, avoidance |
|  | Personal characteristics | character, emotion, life, growth, cognition, conflict, obstacle, age, communication, impression, shadow, avoidance, dominance, character disorder |
|  | Family | parents, mother, family, mom, father, dad, brother, grandmother, sister, daughter, grandparents |
